# A functional hierarchical organization of the protein sequence space

**DOI:** 10.1186/1471-2105-5-196

**Published:** 2004-12-14

**Authors:** Noam Kaplan, Moriah Friedlich, Menachem Fromer, Michal Linial

**Affiliations:** 1 Department of Biological Chemistry, Institute of Life Sciences, The Hebrew University of Jerusalem, Israel; 2 School of Computer Science and Engineering, The Hebrew University of Jerusalem, Israel

## Abstract

**Background:**

It is a major challenge of computational biology to provide a comprehensive functional classification of all known proteins. Most existing methods seek recurrent patterns in known proteins based on manually-validated alignments of known protein families. Such methods can achieve high sensitivity, but are limited by the necessary manual labor. This makes our current view of the protein world incomplete and biased. This paper concerns ProtoNet, a automatic unsupervised global clustering system that generates a hierarchical tree of over 1,000,000 proteins, based solely on sequence similarity.

**Results:**

In this paper we show that ProtoNet correctly captures functional and structural aspects of the protein world. Furthermore, a novel feature is an automatic procedure that reduces the tree to 12% its original size. This procedure utilizes only parameters intrinsic to the clustering process. Despite the substantial reduction in size, the system's predictive power concerning biological functions is hardly affected. We then carry out an automatic comparison with existing functional protein annotations. Consequently, 78% of the clusters in the compressed tree (5,300 clusters) get assigned a biological function with a high confidence. The clustering and compression processes are unsupervised, and robust.

**Conclusions:**

We present an automatically generated unbiased method that provides a hierarchical classification of all currently known proteins.

## Background

The explosive growth in the number of sequenced proteins has created a glut of proteins that are sequenced but whose structure and function are as yet unknown. A common way to tackle this problem is to use database searches to find proteins similar to a newly discovered protein, thus inferring protein function. This method is generalized by protein clustering or classification where databases of proteins are organized into groups or families in a manner that attempts to capture protein similarity. Such classification into families is a critical component in structural and functional genomics [1-4]. The number of protein families comprising the entire protein-space has been conjectured to range between 6,000-30,000, excluding rare and peculiar single proteins [5-8]. Various expert-based databases provide a good description of certain selected families but are limited in scope to thoroughly studied proteins (i.e. [9,10]). Other methods for classification strongly rely on 3D-structural information as in the case of SCOP [11], CATH [12], FSSP [13] and others.

Classifying the entire protein space into families serves not only as a method for large-scale protein annotations but also to support functional and structural genomic initiatives [14]. Some prominent examples for protein classification efforts are ProtoMap [15], Picasso [16], SYSTERS [17], iProClass [18] and ProtoNet [19]. These systems are based on a variety of algorithmic paradigms, each yielding a distinct hierarchical classification of proteins into families.

Amongst the clustering methods listed above only ProtoNet attempts to generate a global hierarchical arrangement of the entire protein space via agglomerative hierarchical clustering. The sequence similarity between every pair of protein sequences is taken as the BLAST [20] E-value between a given pair of proteins. Next, the proteins are clustered using a given merging strategy. The strategy used is Unweighted Pair Group with Arithmetic Mean (UPGMA), whereby in each iteration, the two most similar clusters (in terms of their average pairwise distance for every protein pair spanning the two clusters) are merged. ProtoNet (version 4.0) [21] provides a classification hierarchy of over 1,000,000 proteins including the SwissProt and TrEMBL protein databases [22]. Most proteins included in the SwissProt database are manually validated and furthermore, the degree of biological knowledge associated with them is much higher in comparison to the proteins archived in TrEMBL. Thus, this work concerns only the 114,033 proteins in the SwissProt database (version 40.28). An extended version that includes over one million protein sequences is available in the form of an interactive website at . For the SwissProt-based tree, there are 227,436 clusters (including the proteins as singletons). The classification provided by ProtoNet provides the full range of cluster granularity: from single proteins to huge protein clusters that carry no biological relevance (the root clusters). We test the biological validity of ProtoNet, by its examination from different perspectives, using external-defined protein keyword annotations. Four different annotation sources are used (InterPro [23], GO [24], SCOP [11] and ENZYME [25]) in order to be able to validate different biological aspects. First, we demonstrate that it is possible to match the majority of such external-defined protein families to specific clusters within the ProtoNet clustering. Second, we show that the hierarchy of the ProtoNet tree represents a valid functional hierarchy and correlates well with the GO hierarchical structure.

As mentioned, ProtoNet contains 227,436 clusters, which is obviously much more than the upper estimate of 30,000 protein families [8,26]. Therefore, we seek to cleverly discard those clusters that have less biological relevance. Compression of the protein space offers many advantages. It can yield a smaller set of biologically meaningful clusters, which will allow for a more manageable handling of the entire protein space. Furthermore, if this compression's correspondence to external, independent annotation sources can be validated, then this compression can be used to replace the original hierarchical structure, without sacrificing much information originally present in the whole system.

In this paper we describe methods for the unsupervised compression of the ProtoNet tree, by using intrinsic tree-based parameters of the clusters that correlate well with biological validity. By preserving the unsupervised nature of the ProtoNet data, we prevent biasing towards previously discovered findings and better allow for future generalizations, in addition to maintaining the automation of the process.

Finally, automatic functional annotation to proteins is of great importance. In ProtoNet, an automatic method for assignment of biological annotation to the protein clusters is used, yielding high-confidence functional assignments for a large majority of the proteins' clusters.

## Results and discussion

### Correspondence of clusters to external biological sources

In order to measure the correspondence between a given cluster and a specific annotation, and allow for supervised validation of the ProtoNet clusters, we define the notion of a correspondence score (CS). The CS for a certain cluster and a given keyword measures the correlation between the cluster and the keyword, using the well-known intersect-union ratio.

Let C be a cluster in the ProtoNet tree, and K be a keyword (from a specific source) that annotates (some of) the proteins in the system; Let *c *be the set of annotated proteins in cluster C; Let *k *be the set of proteins in the system annotated by keyword K; We define:


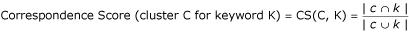


The cluster receiving the maximal score for keyword K is considered the cluster that best represents K within the ProtoNet tree (K's *best cluster*). The score for a given cluster on keyword K ranges from 0 (no correspondence) to 1 (the cluster contains exactly all of the proteins with keyword K, i.e. maximally corresponds to the keyword).

In order to assess the clustering's biological validity, the mean best CS on all annotations was examined for each of the following sources: InterPro, SCOP (Family, SuperFamily, and Fold levels), GO (Molecular Function) and ENZYME (subclass, sub-subclass and entry). The results (Table 1) show a high level of correspondence between the ProtoNet clusters to the various keyword sets of each of the external sources.

It can be argued that a good fit between a set of keywords and the ProtoNet set of protein clusters could happen by chance. In order to assess the statistical significance of these results, the mappings of the keywords to the proteins were randomized, creating a new group of random keyword sets that have the same size distribution but do not represent any biological features. For each random keyword set, the mean best CS was calculated. This randomized test showed a normal distribution, allowing the calculation of an approximate p-value for the mean best CS obtained by ProtoNet for the external sources. The results showed an extremely high level of statistical significance for all sources (all had P-values smaller than 10^-100^). Note that even for the SCOP fold level, which is associated with proteins that may be extremely remote in sequence, ProtoNet's relative success is extremely high (for details on ProtoNet's performance vis-à-vis structural entities, see [27]).

To avoid trivial correspondences between a keyword and a cluster, such as the assignment of a keyword that annotates only one protein to its singleton cluster, we tested our success only with keywords that annotated at least two proteins (for SCOP and ENZYME keywords). For InterPro and GO, we selected a threshold of 20 proteins per keyword, as the majority (85% in InterPro; 98% in GO) of the annotations is included above this threshold, thus allowing the test to focus on the more significant keywords.

### Correspondence of ProtoNet hierarchy to external biological sources

**Figure 1:**
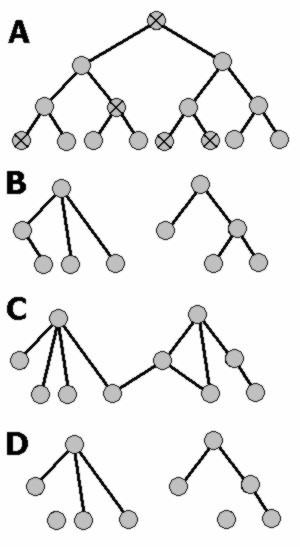
Scheme of the ProtoNet hierarchy test. (A) The ProtoNet binary tree. Vertices in the graph are protein clusters. Crossed-out vertices are eliminated because they do not match any GO term. (B) Tree hierarchy of remaining ProtoNet clusters. Remaining nodes correspond to GO terms. (C) The corresponding GO DAG hierarchy between the vertices. (D) Intersection of graphs in B and C shows the amount of hierarchical correspondence between GO and ProtoNet. In this example, there are 6 edges common to GO and ProtoNet. 2 of the ProtoNet edges do not appear in GO and 4 GO edges do not appear in ProtoNet.

In order to validate the hierarchical structure of ProtoNet, we compare it with the hierarchical structure of GO as described in Figure 1. To do this, we select, for each GO term, the best matching cluster in ProtoNet according to the CS. The subset of all terms that have highly matching clusters (best CS>0.5) was selected. In graph-theoretic terminology, this set of terms can be represented as vertices in a graph. We consider two possible sets of directed edges between the vertices: those defined by GO as the parent-child relationship of the clusters' respective terms, and those of the ProtoNet hierarchy. Thus we wish to compare these two sets of graph edges. We use a very conservative test, counting the number of edges that are common to both graphs.

A total of 1577 GO terms were selected as described, with 1798 edges between them according to the GO hierarchy. 771 out of 1291 (60%) edges that were produced by the ProtoNet hierarchy appear in the GO hierarchy. This number is highly significant considering the fact that there exist over 1,200,000 possible edges between the 1577 vertices in the graph (considering it as a DAG). It should be noted that there are some terms in GO that are connected to many other vertices. These vertices may bias the results of this test. In order to confirm that ProtoNet performs well without these vertices as well, the vertices were removed manually and the test was repeated, with similarly significant results (33% of the edges were correct).

### Compression by using an intrinsic parameter

In order to allow unsupervised automatic compression of the ProtoNet tree, we searched for an intrinsic parameter of the clustering process that would specify clusters of biological validity. By applying such a parameter one could dispose of clusters that do not pass a certain threshold value, remaining with clusters of high biological validity. Once we remain with a subset of biologically valid clusters, the new hierarchy amongst them can be reconstructed according to the original tree hierarchy.

The agglomerative hierarchical clustering scheme defines a set of terms that are intrinsically associated with the process. In such a scheme, each cluster is created from smaller clusters, which are captured as its descendants in the clustering tree. The ProtoLevel (PL) ranges from 0-100 and is used as a standard quantitative measure of the relative height of a cluster in the merging tree. The PL of a cluster is defined as the arithmetic average of the BLAST E-score of the pairs of its proteins. The PL of the leaves of the tree is defined as 0, whereas the PL of a root equals 100. The larger the PL, the later the merging that created the cluster took place. Therefore, the PL scale is considered as an internal monotonic timer of merging, during the clustering process. As mentioned above, a cluster is said to be created when the merging of its two children clusters forms it. The PL at this point is said to be the birthtime of this cluster. The deathtime of a cluster is defined as the PL at its termination, i.e. the point at which it merges into its parent cluster (or 100 if it has no parent). The lifetime (LT) of a cluster is defined as:

LT = deathtime - birthtime

Therefore, the LT of a cluster reflects its remoteness from the clusters in its "vicinity" in protein sequence space.

**Figure 2:**
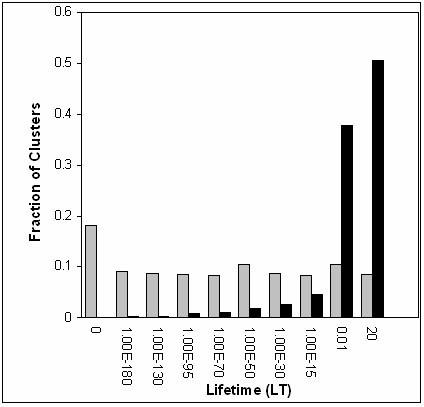
Lifetime (LT) distributions of the set of InterPro best clusters (black bars) in comparison to the LT distribution of all clusters in ProtoNet (gray bars).

We examined the LT distribution of the set of InterPro best clusters in comparison with the LT distribution of all clusters in ProtoNet (Figure 2). The results suggest that the best clusters have a substantially higher LT than other ProtoNet clusters. This poses the LT as a possible candidate that could allow a biologically-valid tree compression by disposing of all clusters with LT below a certain threshold value.

**Figure 3:**
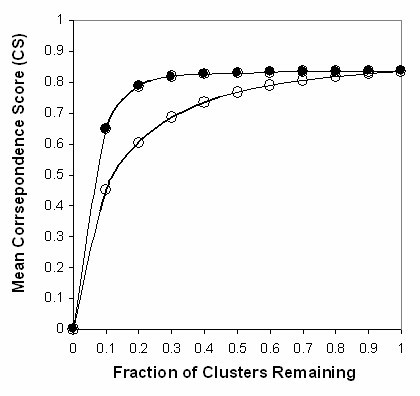
Effect of compression on mean best CS (Correspondence Score) at various threshold values. (A) Mean best CS shown here is calculated for the InterPro keywords. Mean best CS decreases from right to left, as more clusters are eliminated due to compression. Filled circles represent the mean best CS for compression according to LT thresholds. Open circles represent the mean best CS of a random compression to the same extent as explained in the text. Standard deviation of the random mean best CS is too small to be seen on the graph.

In order to search for a reasonable LT threshold value (that would eliminate a large number of clusters while maintaining biological validity), several threshold values were examined (Figure 3). The results show that by using a LT threshold for cluster elimination, in addition to removing the singleton clusters, 87.8% of the clusters may be eliminated with only a minimal reduction in performance (i.e., a reduction of 2.7% in mean best CS), leaving only 27,823 clusters. Furthermore, we compare the LT threshold scheme with a random elimination of similar amounts of clusters. The LT threshold convincingly outperforms the random elimination.

The mean best CS was examined for all four external sources (Table 2). The results show that the mean CS of ProtoNet were only slightly reduced, while the random mean CS are significantly reduced due to the much smaller amount of clusters.

### Automatic functional annotation of clusters

The following scheme was used to annotate the protein clusters: For each cluster C and keyword K we define the following score:


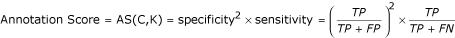


Where *TP *is the amount of true positives (proteins in C that have the keyword K), *FN *is the amount of false negatives (proteins not in C that have the keyword K) and *FP *is the amount of false positives (proteins in C that do not have the keyword K).

For each cluster, we search against all keywords of GO and InterPro for the keyword with the highest AS. If the AS of the cluster is greater than 0.25, the cluster is assigned that keyword as its annotation. The logic behind the score and the threshold is as follows: the score is determined by two parameters, the specificity and the sensitivity; let us consider the two worst-case limit cases. In the first case, specificity>0.5 and sensitivity = 1: a majority of the proteins of the cluster share the keyword, and there exist no other known proteins that have the keyword. In the second case, specificity = 1 and sensitivity>0.25: all proteins of the cluster share the keyword and they constitute more than 1/4 of the total proteins known to have this keyword. In both cases, the keyword can be assigned to the protein cluster with a high degree of confidence. All other clusters fall in between these cases.

**Figure 4:**
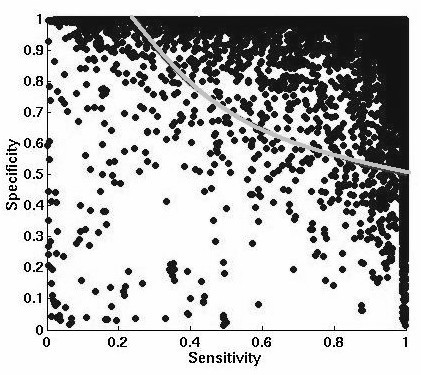
AS (Annotation Score) plot for all clusters of 20 or more proteins, after compression. Each dot represents a cluster and is plotted according to the sensitivity and specificity of its highest-scoring AS (as defined in the text). The curve represents the high-confidence annotation threshold which was used. Dots in the upper right represent clusters that passed the threshold and were therefore annotated with high-confidence.

Using this method, all 6,879 clusters that contain 20 or more proteins and that remain after the compression were tested. 5,355 (77.8%) clusters passed the high confidence threshold and were therefore given an annotation. Figure 4 shows the plot of the highest AS score for each of the clusters and the threshold function. Naturally, by relaxing the threshold it would be possible to obtain a higher level of annotation.

### Cation channels: a biological example

**Figure 5:**
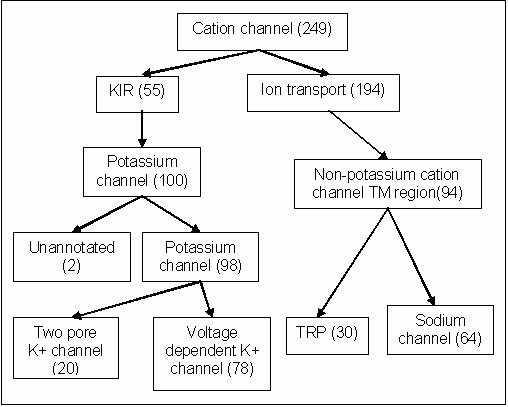
Graph of the ProtoNet tree of cation channels. Squares represent clusters, arrows represent tree hierarchy between clusters. Names are the annotations that were assigned to the clusters as described in text. Parantheses show the number of proteins in the cluster. Note one cluster containing two proteins that was not assigned an annotation.

Figure 5 shows one of the trees that appear in ProtoNet after compression. The root cluster contains 249 proteins and is annotated as "Cation Channel". There appears to be a correct division between potassium channels to non-potassium channels. Furthermore there is an apparent inner division of the potassium channels into two-pore channels and voltage dependent channels, and of the non-potassium cation channels into sodium channels and TRP channels. Notably, an unannotated cluster of 2 proteins is categorized as potassium channel, but does not appear to be voltage-dependent or two-pore. Closer inspection shows that this cluster contains the 2 orthologs of the LctB bacterial protein. Experimental results suggest that LctB is a new type of non-voltage-mediated potassium channel [28]. This corresponds well to the fact that ProtoNet did not assign an annotation to this cluster and separated it from the other potassium channels.

## Conclusions

The challenge of protein classification by using sequence similarity has been addressed extensively by several different methods. In order to assign function to proteins, advanced methods (such as Hidden Markov Models implemented in Pfam) have been used to learn sequence-based patterns on "seeds", manually validated alignments of known protein families. The widely-used BLAST algorithm is considered to be a reliable tool for sequence alignment, but has been shown to lack sensitivity for weak similarities that may be detected by signature detection methods. We show here that by using an unsupervised bottom-up clustering method based on BLAST E-values, we have been able to construct a global hierarchy of the SwissProt proteins that can be validated by external biological sources, merely by undertaking a global view of the protein space.

The four different external sources that were used for validation reflect different aspects of the protein space: InterPro annotation is predictive and is based on various signature detection methods; GO annotation assignments are both based on prediction and from known research, while the GO hierarchy was constructed completely manually; SCOP is a semi-manual classification of structures that is not necessarily reflected in sequence; the ENZYME database supplies Enzyme Commissions, which constitute a hierarchy that is based on the enzymes' chemical reactions. ProtoNet successfully constructs clusters that correspond highly to all four of the sources. Even high levels of SCOP (such as the Fold classification), which are considered to have no detectable sequence similarity, are partially matched (also see discussion in [27]). Notably, the correspondence of ProtoNet to InterPro is generally higher than the correspondence to the other sources. This is not surprising, considering the fact that InterPro is based on prediction from sequence. However, it is worthwhile to note that the InterPro families may be reconstructed almost perfectly without using the various sensitive detection methods that InterPro uses, and more importantly without using the manually constructed seeds.

After validating the biological relevance of the ProtoNet clusters by using external sources, we examined the hierarchy of ProtoNet. The test showed that the hierarchy presented by ProtoNet significantly corresponds to the manually-constructed biological hierarchy of GO. It is important to note that the method used by ProtoNet is not expected to fully recapture the GO hierarchy due to the fact that ProtoNet is structured as a collection of trees while GO is structured as a DAG. In this sense, the approach of ProtoNet may be limited in the portrayal of evolutionary complexity (as in cases of multiple domains). However by using a domain-based clustering approach, allowing multiple entities of each protein in the hierarchy, a substantial improvement in the CS quality measure may be achieved (unpublished results).

An intrinsic parameter that reflects the stability of clusters during the clustering process was used in order to compress the cluster sets, leaving 16.4% of the clusters; removing the singletons clusters as well leaves 12.2% of the clusters. As mentioned above (see Methods), the entire ProtoNet scaffold contains 227,436 clusters that are represented by 630 roots; following this condensation, there are only 27,823 clusters that are represented by 2,236 roots. We show that although a massive portion of the clusters is discarded, very little performance is lost by this condensation process. It is obvious that prior to the condensation process, ProtoNet holds within it both clusters that correctly represent biological groups and clusters that are irrelevant artifacts of the clustering process (e.g. the large root clusters that are constituted of tens of thousands of proteins). Therefore, by allowing a major reduction without significant loss of biological coherence ProtoNet seems to present a more correct view of the protein world.

An automatic unsupervised method for the classification of proteins has some important advantages over supervised methods (such as signatures based on manually validated seeds): First, an unsupervised method is unbiased in automatic assignment of function to proteins, a major goal in bioinformatics. Also, it allows high-throughput analysis of whole genomes and enhances understanding of global biological systems without the need for the manual annotation of every protein. Using an automatic annotation method, we are able to successfully annotate 77.8% of the major protein clusters (of size 20 or more) that remain after the compression of the ProtoNet tree. The annotation uses a relatively conservative threshold and therefore yields high-confidence annotations. This further suggests that the clusters remaining after the condensation process are relevant biological clusters and not mere artifacts.

One aspect that we have rigorously examined is the robustness of the ProtoNet tree: given a different set of proteins to cluster or a different clustering method, would the resulting tree be significantly different, or are its properties maintained? Interestingly, changing the underlying protein databases (ranging in size from 30,000 to over 1,000,000 proteins), the substitution matrices used for the preliminary BLAST, or the merging strategy [19] produced very similar trees (unpublished results), suggesting that the performance of ProtoNet is not due to a specific computational method but perhaps to the robust properties of the protein sequence space.

## Methods

ProtoNet version 2.4 which was used for the analyses described in this paper is based on classification of the SwissProt database (version 40.28) that contains 114,033 proteins. The entire ProtoNet scaffold contains 227,436 clusters that are contained in 630 trees. Most trees (611) are singletons and only one contains most (>99%) of the proteins. For more details on the construction of the ProtoNet hierarchy see [19]. ProtoNet version 4.0 [21] which is available online contains a wider classification of over 1,000,000 proteins (a union of the SwissProt and TrEMBL databases).

Several external sources were used as a biological reference for validation of the ProtoNet tree: InterPro [23] is an extensive family and signature archive that integrates several different databases: PRINTS, Pfam, PROSITE, ProDom, Smart, TIGRFAMs, and recently also PIR SuperFamily and SUPERFAMILY. Each of these databases relies on a different detection method. Many of these signatures and family keywords are considered to be undetectable by a routine BLAST search. InterPro (version 5.2) contains 5,551 signatures. Gene Ontology (GO) [24] is a collaborative project of creating a hierarchy of biological terms. GO is represented as a directed acyclic graph (DAG), which is divided into three parts: Molecular Function, Cellular Localization and Cellular Process. In this study only the Molecular Function aspects of GO were used. GO's Molecular Function subdivision (July 2002) contains 5,947 biological terms. SCOP [11] is a hierarchical representation of protein structures. SCOP uses a tree-like hierarchy of 4 levels: Class, Fold, SuperFamily and Family. SCOP (version 1.57) contains 2,927 structures terms. The ENZYME database (as part of Swissprot data) indicates the EC number of a protein [25]. EC (Enzyme Commission) numbers are a classification scheme for enzymes, based on the chemical reactions they catalyze. The EC number includes 4 fields (for example, 1.2.3.4 represents the enzyme class, subclass, sub-subclass and entry number, respectively). ENZYME (updated July 2002) contains 3,958 enzyme classifications.

We have used EBI mappings of InterPro and GO to SwissProt proteins.

## List of abbreviations

Annotation Score (AS), Correspondence Score (CS), Directed Acyclic Graph (DAG), Enzyme Commission (EC), Gene Ontology (GO), Lifetime (LT).

## Authors' contributions

ML conceived of the compression of the ProtoNet tree. NK, MF and MF participated in implementation of the condensation and in the design and implementation of the various tests. All authors participated in the analysis of the results. All authors read and approved the final manuscript.

## Figures and Tables

**Table 1:** Correspondence of external biological keywords and ProtoNet clusters.

**External Source**	**ProtoNet Mean Best CS^a^**	**Random Mean Best CS (std dev)**	**# KW**
InterPro	0.835	0.026 (0.9*10^-4^)	2034
GO molecular function	0.588	0.024 (0.8*10^-4^)	1220
SCOP family	0.720	0.299 (0.8*10^-4^)	742
SCOP superfamily	0.654	0.260 (0.9*10^-4^)	558
SCOP fold	0.598	0.230 (1*10^-4^)	408
ENZYME entry	0.848	0.179 (0.7*10^-4^)	1432
ENZYME sub-subclass	0.517	0.053 (2*10^-4^)	161
ENZYME subclass	0.412	0.025 (4*10^-4^)	56

^a^CS – correspondence score, see text for definition.

**Table 2:** Correspondence of external biological keywords and ProtoNet clusters after and before compression.

**External Source**	**ProtoNet Mean CS (before compression)^a^**	**Random Mean CS (before compression)**
InterPro all	0.808 (0.835)	0.025 (0.026)
GO molecular function	0.558 (0.588)	0.020 (0.024)
SCOP family	0.702 (0.720)	0.124 (0.299)
SCOP superfamily	0.635 (0.654)	0.116 (0.260)
SCOP fold	0.580 (0.598)	0.107 (0.230)
ENZYME entry	0.643 (0.848)	0.090 (0.179)
ENZYME sub-subclass	0.471 (0.517)	0.036 (0.053)
ENZYME subclass	0.371 (0.412)	0.018 (0.025)

^a^CS – correspondence score, see text for definition.
